# Iron and zinc homeostasis in plants: a matter of trade-offs

**DOI:** 10.1093/jxb/erad304

**Published:** 2023-09-29

**Authors:** Marc Hanikenne, Frédéric Bouché

**Affiliations:** InBioS-PhytoSYSTEMS, Translational Plant Biology, University of Liège, B-4000 Liège, Belgium; InBioS-PhytoSYSTEMS, Translational Plant Biology, University of Liège, B-4000 Liège, Belgium; InBioS-PhytoSYSTEMS, Plant Physiology, University of Liège, B-4000 Liège, Belgium

**Keywords:** Deficiency, excess, iron, regulation, signalling, toxicity, zinc

## Abstract

This article comments on:

Stanton C, Rodríguez-Celma J, Krämer U, Sanders D, Balk J. 2023. BRUTUS-LIKE (BTSL) E3 ligase-mediated fine-tuning of Fe regulation negatively affects Zn tolerance of Arabidopsis. Journal of Experimental Botany 74, 5767–5782.

**Iron and zinc are essential micronutrients in plants, which have evolved homeostatic mechanisms to ensure a proper balance between these two transition metals. However, zinc excess causes toxicity symptoms linked to secondary iron deficiency in shoots of Arabidopsis. Stanton *et al.* (2023) show that these symptoms are attenuated in a *btsl1*/*btsl2* (*BRUTUS-LIKE E3 ligase*) double mutant, which is defective for negative regulation of the iron deficiency response believed to protect the plant against the deleterious effect of iron overload. These findings suggest that a trade-off exists between ensuring proper iron nutrition and protecting against zinc excess in Arabidopsis.**

Iron and zinc are essential micronutrients involved in a wide range of cellular functions (Box 1; Clemens, 2021; Rengel *et al.*, 2023). Both metals are poorly available in soils and in the starch-rich endosperm of cereal grains (Rengel *et al.*, 2023), which causes mild to severe iron and zinc deficiency in >2 billion people worldwide. The greatest impact is on children living in poverty and populations relying almost solely on plant-based diets, thus leading to major health issues that adversely affect education and economic development in those regions (Assunção *et al.*, 2022).

**Box 1. The multifaceted roles of iron and zinc in plants**

Although iron is the fourth most abundant element in the Earth’s crust, it is poorly available in soils, in particular at more alkaline pH. Iron is a transition metal that: (i) can accommodate a variable number of electrons in the d shell, thus allowing, in ionic form, two easily interconvertible oxidation states [Fe(III) and Fe(II), 5 and 6 d shell electrons, respectively] in biological systems; and (ii) can form octahedral complexes with various ligands. Iron has major roles in biological redox systems, mostly as a cofactor of multiple proteins (~2% of all proteins). Given its chemical properties, the reactivity of iron in its ionic form must be tightly controlled in cells to avoid unwanted reactions and oxidative damage, and it is therefore chelated within cells or for long-distance transport in the plant. Iron, as Fe–S clusters or haem, is a key component of electron transport chains in the chloroplast and in the mitochondria, therefore being crucial for photosynthesis and respiration, respectively. It is estimated that up to 80% of the cellular iron in leaf cells is found in chloroplasts. In addition to electron transport, different types of iron cofactors also mediate a range of catalytic reactions, for example in carbon metabolism, and nitrate and sulfate assimilation. It is estimated that 50–250 μg Fe g^−1^ leaf dry weight is the optimal physiological iron concentration in most plants.

Zinc is a metallic element with a d shell fully filled with 10 electrons and, in ionic form, exists only in one oxidation state [Zn(II)]. In organisms, it is the second most abundant transition metal after iron. Like iron, its availability in soils decreases at higher pH. The chemical properties of zinc are reflected in its functions in biological chemistry, which are of two main types. First, as Zn(II) is a strong Lewis acid with flexible geometry and fast ligand exchange, it is used for catalysis of hydrolytic and oxidoreductase reactions. It is the only metal present in all six of the major classes of enzymes, including enzymes such as carbonic anhydrase and the copper/zinc superoxide dismutase (Cu/ZnSOD). Second, as a strong binder devoid of redox activity, zinc is also abundantly used for structural functions within proteins and in protein–protein interactions, or to interact with nucleic acids without producing oxidative damage. For instance, zinc-finger transcription factors and other nucleic acid-interacting proteins contain zinc as a structural ligand. It is estimated that zinc is used as a ligand by >10% of proteins, the so-called zinc proteome. Given its abundance in the proteome, zinc is therefore involved in multiple key functions in cells. In most plants, it is estimated that 30–200 μg Zn g^−1^ dry weight is the optimal physiological zinc concentration.

## Interactions between iron and zinc homeostatic networks

Iron and zinc contents in edible plant parts are controlled by complex and intertwined homeostasis mechanisms involving multiple steps (Hanikenne *et al.*, 2021; Riaz and Guerinot, 2021; Stanton *et al.*, 2022; Vélez-Bermúdez and Schmidt, 2023). The uptake of iron and zinc by roots starts with their mobilization in the soil and their transport across the plasma membrane of epidermis cells. This is followed by radial transport towards the root stele, whose rate is determined by a balance between radial movement and retention in root vacuoles. Translocation to the shoot then requires transporters for xylem loading as well as metal binding to chelators to ensure their movement in the evapotranspiration stream. Additional players are subsequently required for metal unloading, trafficking, and distribution to cells and organelles in shoot tissues and, upon flowering, for phloem-based distribution of metals to reproductive tissues (Olsen and Palmgren, 2014; Amini *et al.*, 2022). Although iron and zinc fulfil very distinct functions in cells (Box 1), interactions between their homeostatic networks exist and stem from similar ionic radii and coordination geometries, which results in: (i) possible mismetallation of enzymes and proteins; and (ii) shared transporters and chelators (Hanikenne *et al.*, 2021). For instance, zinc excess in Arabidopsis (*Arabidopsis thaliana*) results in strong iron deficiency symptoms in shoots, which are alleviated by increased iron supply (Hanikenne *et al.*, 2021). Furthermore, the regulation of IRT1 (IRON-REGULATED TRANSPORTER 1), the main iron uptake transporter in Arabidopsis roots, not only controls iron availability in tissues but also has a built-in mechanism to ensure a proper balance between iron uptake and excessive uptake of other divalent metals (Box 2; Dubeaux *et al.*, 2018; Spielmann and Vert, 2021).

**Box 2. A brief overview of the regulation of the iron deficiency response in Arabidopsis**

**Fig. 1.**
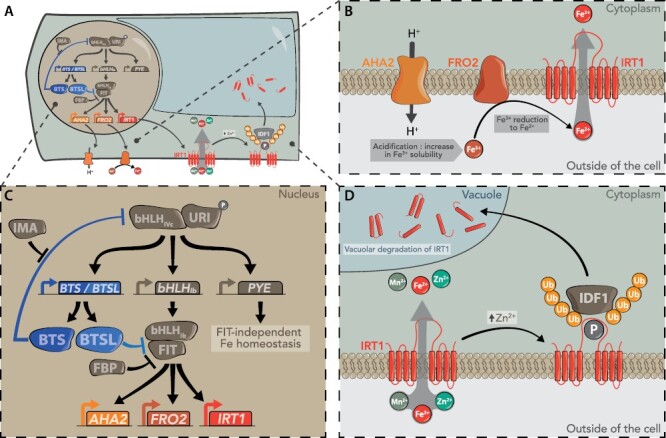
A simplified view of the regulation of the iron deficiency response (A–C) and of the post-translational regulation of the IRT1 transporter (A, D) in Arabidopsis. See the text of Box 2 for details.

Upon iron deficiency, the transcriptional induction of the main players in iron uptake in the roots, a proton pump (*AHA2*), a ferric chelate reductase (*FRO2*), and an iron transporter (*IRT1*), is placed under the control of a IIIb bHLH (basic helix–loop–helix) transcription factor called FIT (Fig. 1A–C). FIT is active when it forms heterodimers with Ib bHLHs (bHLH38, bHLH39, bHLH100, and bHLH101). The *Ib bHLH* genes are themselves up-regulated upon iron deficiency by other bHLH proteins acting as heterodimers, URI (UPSTREAM REGULATOR OF IRT1 or bHLH121, IVb subgroup) and IVc bHLHs (bHLH34, bHLH104, bHLH115, and bHLH105). URI and IVc bHLHs also regulate: (i) Popeye (PYE, a IVb bHLH), which suppresses iron storage in ferritin (independently of FIT); and (ii) BTS and BTSLs. When iron is present, BTS is thought to target IVc bHLH for degradation by the 26S proteasome, whereas BTSLs quickly inactivate FIT when iron is resupplied after a period of iron deficiency. IMA peptides themselves block the action of BTS/BTSLs when iron is limiting. FBP (FIT-binding protein) is another repressor of FIT, acting by binding to its DNA-binding site in the root stele when iron is sufficient. *bts*, *btsl1/btsl2*, and *fbp* mutants, all impaired in inactivating FIT, display zinc tolerance. Adding further complexity to this multilayered regulatory pathway, FIT also acts as a hub to integrate multiple cellular signals (calcium, ethylene, gibberellins, jasmonic acid, and reactive oxygen species) upon iron deficiency.

The IRT1 protein is the main Fe(II) transporter in roots. IRT1 is subject to complex post-translational regulation to ensure the uptake of the right amount of iron, with limitation and excess both being toxic, and to deal with its broad specificity for divalent metal cations (e.g. zinc and manganese). In control conditions, internalization of IRT1 from the plasma membrane to internal membranes is driven by multi-monoubiquitination, with a proportion of the IRT1 protein then being sent to the vacuole for protease-mediated degradation while another proportion is recycled to the plasma membrane (Fig. 1A, D). Binding of excess zinc or manganese to a histidine-rich cytosolic loop of IRT1 leads to the recruitment of the IDF1 E3 ubiquitin ligase, the polyubiquitination of IRT1, and its internalization, targeting to the vacuole and degradation, thus preventing toxic metal accumulation.

In Arabidopsis, genetic work has identified several transporters and metal chelators contributing to both iron and zinc homeostasis [see Hanikenne *et al.* (2021), and the summary in Stanton *et al.* (2023)]. In a straightforward pathway, F-bZIP (F-group BASIC LEUCINE-ZIPPER) transcription factors are activated by zinc deficiency to transcriptionally induce zinc uptake transporters (Lilay *et al.*, 2021) while a multilayered and complex regulatory pathway involving transcription factors, regulators, and signalling processes controls the iron deficiency response (Box 2; Riaz and Guerinot, 2021; Vélez-Bermúdez and Schmidt, 2023). In contrast, little is known about the regulation of the interactions between the iron and zinc homeostatic networks.

## BRUTUS and BRUTUS-like proteins at a crossroad between iron and zinc homeostasis pathways

The ubiquitin E3 ligase BTS (BRUTUS) together with the closely related BTSL1 and BTSL2 (BTS-Like) proteins are negative regulators of the iron deficiency response (Box 2). They catalyse the ubiquitination of key transcription factors, hence targeting them for degradation upon sufficient iron supply (Hindt *et al.*, 2017; Rodríguez-Celma *et al.*, 2019). BTSL1 and BTSL2 are particularly important to shut down the iron deficiency response when iron is resupplied after a deficiency period (Rodríguez-Celma *et al.*, 2019). Such a response is instrumental to prevent iron overload. Accordingly, the corresponding *bts* and *btsl1/btsl2* mutants are more tolerant to iron deficiency, more sensitive to iron excess, and accumulate moderate (*btsl1*/*btsl2*) to high (*bts*) levels of iron when grown in control conditions (Hindt *et al.*, 2017; Rodríguez-Celma *et al.*, 2019).

Recently, several studies have reported that the aforementioned mechanisms repressing the iron deficiency response also influence zinc tolerance (Chen *et al.*, 2018; Zhu *et al.*, 2022; Stanton *et al.*, 2023). *bts* and *btsl1/btsl2* mutants were shown to be more zinc tolerant, although this phenotype was accompanied by distinct alterations of their ionome (Zhu *et al.*, 2022; Stanton *et al.*, 2023). For the *btsl1/btsl2* double mutant, root and shoot zinc tolerance phenotypes were dependent on the iron:zinc ratio provided in the growth medium. Using carefully designed experiments, including ionome and transcriptomic profiling as well as split-root experiments, Stanton *et al.* (2023) started to unveil the underlying mechanisms. The massively increased iron accumulation in roots upon zinc excess that is observed in wild-type Arabidopsis plants was almost fully abolished in the *btsl1/btsl2* double mutant. Unexpectedly, the FIT (FER-like IRON DEFICIENCY INDUCED TRANSCRIPTION FACTOR)-dependent and independent iron deficiency responses, which regulate iron uptake and iron chelation/storage, respectively (Box 2), were more strongly induced in the mutant roots. In stark contrast, the iron deficiency transcriptional response observed in wild-type shoots upon zinc excess was totally absent in the mutant, including the induction of IMA (IRON MAN) signalling peptides (Box 2). IMA peptides, which are known to play a role as a systemic iron deficiency signal emitted by shoots through the phloem, also interact with BTS and BTSLs to prevent their repressive action, thus enabling a sustained iron deficiency response (Li *et al.*, 2021; Lichtblau *et al.*, 2022). Split root experiments further suggested that the action of BTSL1 and BTSL2 on zinc tolerance occurred locally in roots and downstream of systemic IMA signalling (Stanton *et al.*, 2023). Altogether, increased shoot zinc tolerance, a higher iron:zinc ratio in shoots, and reduced expression of iron deficiency genes in the *btsl1/btsl2* double mutant are consistent with reduced iron deficiency, and hence zinc toxicity in shoots (Stanton *et al.*, 2023). Based on these observations, Stanton *et al.* (2023) propose a model in which, despite the opposite action of zinc excess and iron deficiency on metal uptake by IRT1, the *btls1/btls2* mutation enables a moderate but sufficient increase of iron uptake to counterbalance the toxicity of zinc excess. In contrast, when exposed to zinc excess, wild-type plants encounter strong iron deficiency in shoots, which signals a strong and constitutive iron deficiency response in roots, similar to what is observed in an *frd3* mutant (Durrett *et al.*, 2007; Scheepers *et al.*, 2020). The negative action of BTLS1 and BTSL2 on FIT, which prevents excessive and toxic uptake of iron, would thus result in increased zinc toxicity. Future work will be needed to examine the contribution of IRT1 internalization to the crosstalk described by Stanton *et al.* (2023). How the loss of BTLS1/BTSL2 results at the same time in reduced iron uptake by roots and in sufficient iron supply to shoots in the *btls1/btls2* background will also require further examination.

## The imperfect nature of metal homeostatic networks

The recent advances in the characterization of metal homeostasis mechanisms in plants unravel an imperfect landscape shaped by trade-offs. This complexity is highlighted in the study of Stanton *et al.* (2023) on BTSL1 and BTSL2, as well as for the *frd3* mutant, whose striking root growth defect is partially alleviated by zinc (Scheepers *et al.*, 2020). Another example is IRT1, which, as a key transporter for iron uptake, requires very complex post-translational regulatory mechanisms to rein in its ability to transport other divalent metal cations (Box 2; Dubeaux *et al.*, 2018; Spielmann and Vert, 2021). That a more specific version of IRT1 did not evolve over time suggests that in certain circumstances, the poor selectivity of this protein must be advantageous and/or that tightly regulating its transport activity provides fine-tuning of metal uptake. IRT1 is indeed thought to contribute substantially to intraspecific variation in zinc/cadmium or nickel hyperaccumulation among populations in several metallophyte species (Merlot *et al.*, 2021). Similarly, a recent study showed that the constitutive high *ZIP6* expression observed in the zinc and cadmium hyperaccumulator plant *Arabidopsis halleri* in fact causes an increase in cadmium toxicity, and suggested that the action of the gene results in a trade-off between toxic uptake of cadmium and possibly metal distribution between tissues of the plant (Spielmann *et al.*, 2020).

These examples converge toward the fact that such trade-offs are necessary to fine-tune the metal homeostatic networks which, like all homeostatic mechanisms, can be overcome or become dysfunctional when pushed to extremes, such as when wild-type Arabidopsis plants (with functioning BTSL1/BTSL2 genes) are confronted i.e. with a very high zinc:iron supply ratio (Stanton *et al.*, 2023).

## Perspectives

In their natural environment, plants are likely to encounter wide variations in nutrient supply and can be exposed to many combinations of nutrient excess or limitation. In recent years, plant nutrition research has therefore moved from studying the role of single nutrients to the exploration of the interactions between nutrients, thus identifying shared players and tracking the regulating, sensing, and signalling mechanisms involved in these interactions. The study by Stanton *et al.* (2023), together with recent and complementary work (e.g. Zhu *et al.*, 2022), sheds light on how the interactions between iron and zinc are integrated into the complex iron regulatory network (Box 2). Moreover, major progress has also been recently achieved in our understanding of the interactions of iron or zinc with phosphate (Hanikenne *et al.*, 2021; Lay-Pruitt *et al.*, 2022). How regulators of the iron deficiency response, such as BTS and BTSLs, intervene in the ‘*ménage à trois*’ between iron, zinc, and phosphate is an important question to address in future research. A precise understanding of these interactions will be key to design meaningful strategies to optimize sustainable crop production in the context of limited resources and global change.
